# Carotid Sinus Syndrome With Convulsive Syncope in a Patient With Head and Neck Cancer

**DOI:** 10.7759/cureus.62032

**Published:** 2024-06-09

**Authors:** Dosbai Saparov, Mustafa Wasifusddin, Narek Hakobyan, Lena Khachik, Rukhsana Aslam

**Affiliations:** 1 Internal Medicine, Brookdale University Hospital Medical Center, Brooklyn, USA

**Keywords:** head and neck cancer (h&n cancer), carotid sinus hypersensitivity, palliative radiation therapy, carotid sinus syndrome, tachy-brady syndrome, recurrent syncope

## Abstract

This case report provides a comprehensive overview of a unique case of a 64-year-old male patient with head and neck (H&N) cancer who initially presented with compressive convulsive syncope, an initial manifestation of carotid sinus syndrome (CSS). CSS is an autonomic nervous system disease that often manifests as hypotension, dizziness, cerebral ischemia, or syncope, usually in elderly patients. In this case, the patient's laryngeal cancer led to lymphedema and encasement of the bilateral carotid arteries, inducing CSS and resulting in recurrent episodes of hypotension and bradycardia. These symptoms were managed through the administration of atropine and transcutaneous pacemaker placement, suggesting a probable mixed type of CSS. The patient was discharged on long-term theophylline treatment for symptomatic control of bradycardia episodes. Despite the promising outcomes of CSS cases treated with pacemakers, the efficacy is not universal and limitations may arise, particularly in H&N cancer patients. Therefore, the patient was managed with theophylline rather than a pacemaker due to its non-invasiveness and effectiveness in temporarily managing CSS. Although rare, CSS should be considered in patients experiencing convulsive syncope alongside H&N malignancies. As the evidence and consensus regarding CSS treatment in H&N cancer patients are scarce, additional research is necessary to evaluate and compare available options. This abstract concludes by emphasizing the need for further research and case reports to establish a consensus on the optimal management approach for patients affected by CSS due to compression from H&N cancers.

## Introduction

The carotid sinus is a neurovascular dilation located at the base of the internal carotid artery, just distal to the bifurcation of the common carotid artery. It is comprised of specialized sensory nerve endings known as baroreceptors, which can detect fluctuations in blood pressure and initiate appropriate compensatory responses via the carotid sinus reflex [1]. As blood pressure rises, stretching of the arterial wall at the carotid sinus stimulates baroreceptors. Afferent signals are transmitted via the nerve of Hering, a branch of the glossopharyngeal nerve, to the solitary tract nucleus at the medulla. Subsequently, efferent signals are transmitted to the heart and blood vessels via sympathetic and parasympathetic fibers. This triggers changes in the heart rate and peripheral vascular tone to modulate autonomic outflow.

Carotid sinus hypersensitivity (CSH) is an exaggerated form of the carotid sinus reflex, characterized by a heart rate pause lasting longer than three seconds and a systolic blood pressure drop greater than 50 mmHg [2]. Many individuals with CSH may remain undiagnosed as they are often asymptomatic. However, in some cases, CSH can manifest as carotid sinus syndrome (CSS). CSS presents with symptoms, such as hypotension, cerebral ischemia, dizziness, or syncope [3]. Triggers for CSS commonly include neck movement, shaving, coughing, sneezing, wearing tight neck collars, carotid sinus massage, or compression. Rarely, head and neck (H&N) tumors can present as CSS [3]. As these tumors progress, they may encroach upon the carotid sinus, impinge on the glossopharyngeal nerve, or directly exert pressure on the carotid body. 

Syncope is a frequently encountered reason for hospital admission with a wide range of potential diagnoses [4,5]. Patients presenting with syncope should be placed on telemetry for constant EKG monitoring. Patients with syncope and episodic bradycardia should undergo investigation for any compression of the carotid sinus [6]. Notably, cases linking H&N cancers to syncope and seizure-like episodes upon initial presentation are exceptionally rare, with existing data documented only in the form of case reports [7]. Herein, we report a unique case of a patient with an H&N cancer who presented with an initial manifestation of compressive convulsive syncope.

## Case presentation

A 64-year-old male patient with a past medical history of hypertension and gastroesophageal reflux disease, chronic obstructive pulmonary disease, and a social history significant for alcohol consumption on weekends and smoking for more than 40 packs-years (currently actively smoking), was taking pantoprazole at home. He presented to the hospital for an unwitnessed fall followed by a seizure witnessed by his daughter at home. He had a month-long history of progressive bilateral neck swelling and dysphagia to both solids and liquids. He also reported night sweats and weight loss over the last year, but he was unable to approximate the exact amount. He noted that he was feeling dizzy and had some shortness of breath prior to his fall. He had been experiencing shortness of breath at times but was unable to specify when it began.

**Figure 1:**
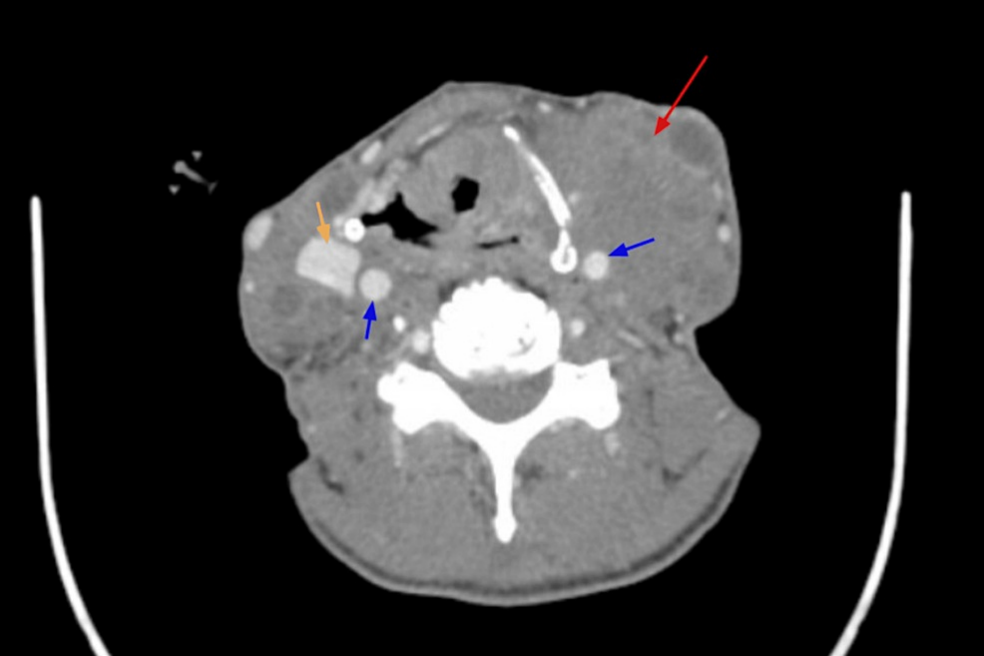
CT scan of the neck with contrast (axial view): large mass (red arrow) near the carotid artery (blue arrows). The internal jugular vein (orange arrow) is seen on the right side.

**Figure 2:**
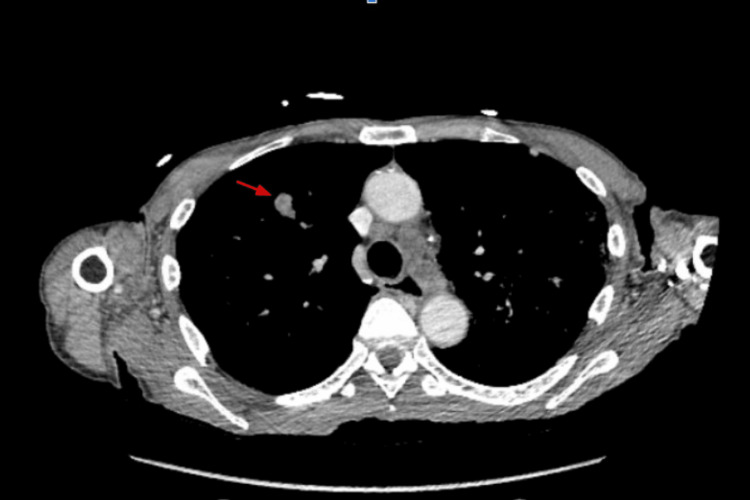
CT of the chest with contrast (axial view): pulmonary nodule likely representing metastatic disease (red arrow)

Upon investigation, his blood pressure (BP) was 127/75 mmHg, heart rate (HR) was 56 beats per minute, respiratory rate (RR) was 20, and oxygen saturation (SpO_2_) was 100% on room air. The physical exam revealed significant lymphadenopathy over the bilateral submandibular and anterior and posterior cervical chain. Some lymph nodes were soft and mobile, while others were firm and immobile. There were no significant electrolyte abnormalities, except for hypomagnesemia (1.7), which was replaced and corrected afterward. CT imaging of the soft tissue neck with contrast revealed encasement of the carotid vasculature with the inability to view the left internal jugular vein, possibly due to occlusion or compression (Figure 1). Possible invasion of this process into the left supraglottic tissues with possible partial destruction of the left side of the thyroid cartilage and airway deviation to the right was also seen. CT imaging of the chest showed multiple pulmonary nodules of the upper lobes and lower lobes with aortopulmonary window and right hilar and azygoesophageal recess adenopathy (Figure 2). Correlating the imaging with the patient's history suggests possible metastatic disease to the lungs. MRI imaging of the brain did not reveal any signs of metastasis.

**Figure 3:**
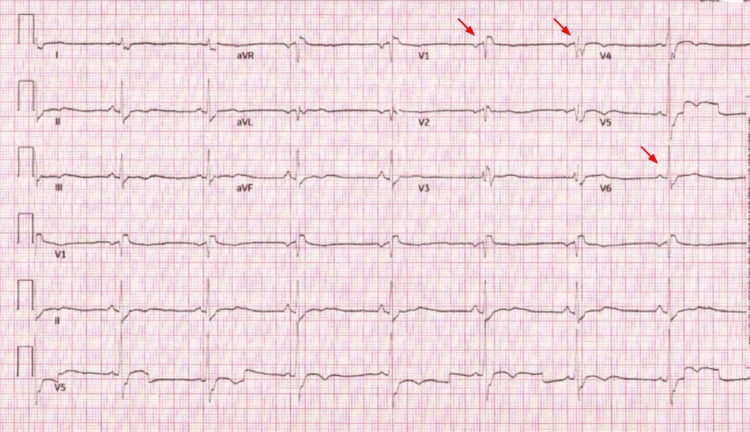
EKG showing sinus bradycardia (heart rate is 48) with a right bundle branch block (red arrows in V1 and V6 leads) before atropine administration

During his hospital stay, he experienced an additional episode of syncope while undergoing a bedside left carotid duplex with an EKG showing sinus bradycardia (Figure 3). He was transferred to telemetry and evaluated for carotid sinus hypersensitivity as the etiology of the syncope. In telemetry, he was noted to have an episode of symptomatic bradycardia, hypotension, altered mentation, and sinus pauses of greater than three seconds. At that time, his blood pressure was 65/40 mmHg, MAP was 49, and heart rate was 40 beats per minute (BPM), and he received only 1 liter of NS bolus. After his BP normalized quickly(BP was 131/77), but bradycardia persisted (HR was 43-49). Simultaneously, transcutaneous pacing at 60 bmp 30mA was started for him at that time due to his hemodynamic instability. At this point, he was transferred to the cardiac care unit (CCU) for the management of symptomatic bradycardia and syncope. 

**Figure 4:**
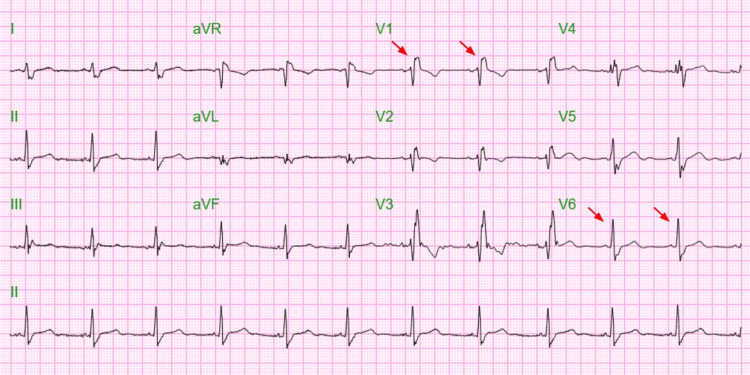
EKG showing normal sinus rhythm (heart rate is 67) with right bundle branch block (red arrow in V1 and V6) after atropine administration

In the CCU, he was given 1 mg of atropine with a noted improvement in his heart rate (Figure 4). His transcutaneous pacing was continued with a threshold of 60 bpm. During his CCU course, he experienced several episodes of bradycardia in relation to head movement and carotid involvement. Hence, a neck collar was recommended. He also had an episode of myoclonic jerking of his extremities lasting less than 10 seconds. The patient was then started on theophylline to prevent periods of bradycardia triggered by carotid body stimulation and continued wearing the neck collar to prevent carotid body stimulation. Subsequently, he did not experience hypotensive episodes or sinus pauses on the cardiac monitor.

**Figure 5:**
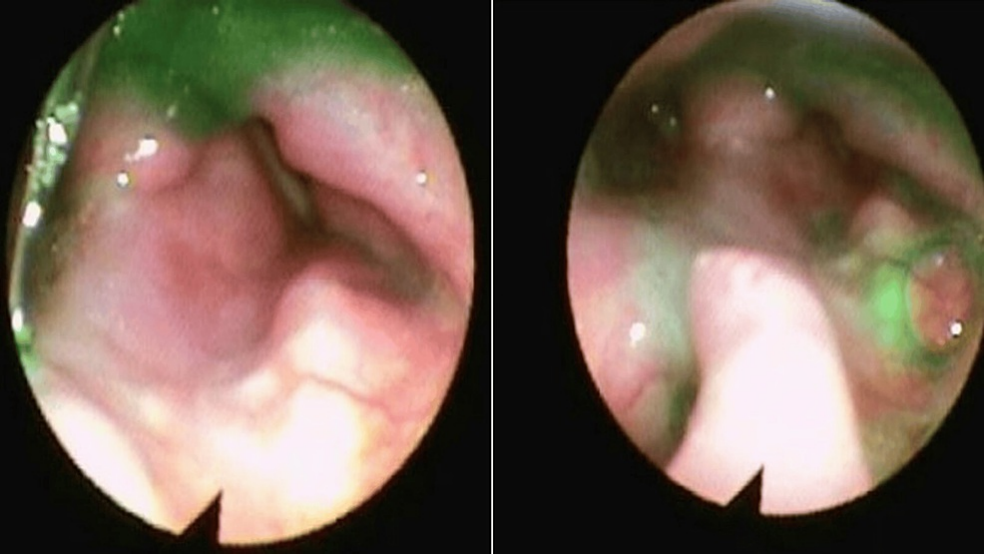
Laryngoscopic view of a laryngeal mass in the left supraglottic region with moderate narrowing of the airway

An ultrasound-guided lymph node biopsy revealed metastatic squamous cell carcinoma, p40+, p16+, CK5/6+, CK7-, and EMA-. The carotid sinus hypersensitivity was attributed to the encasement of the carotid artery by the tumor. After consulting hematology-oncology, the decision was made to treat with palliative radiotherapy only at this time. A dose of 5000 cGy was scheduled for 20 sessions (250 cGy x 20). After receiving six sessions, his bradycardia symptoms resolved. He did not experience episodes of bradycardia, hypotension, or sinus pauses when turning his head or while sleeping. Chemotherapy was deferred to avoid exacerbation of the edema from therapy that could further irritate the carotid body and thereby worsen the patient's bradycardia or hypotension. Theophylline was used to achieve near-total symptomatic control. A few months following, the patient was started on first-line therapy including pembrolizumab, carboplatin, and 5-FU with G-CSF therapy. Because of the obstruction caused by the mass, the patient experienced significant difficulty swallowing and changes in voice quality (Figure 5). Subsequently, a percutaneous endoscopic gastrostomy (PEG) tube was inserted to ensure adequate nutrition and hydration. 

Following the completion of 20 sessions of RT (total 5000 cGy) and the first cycle of chemotherapy with pembrolizumab 200 mg, carboplatin 450 mg, and 5-fluorouracil 5,750 mg, there was a notable reduction in the size of the laryngeal mass. Consequently, the patient demonstrated improved swallowing function and was able to tolerate a pureed diet.

## Discussion

CSS is an autonomic nervous system disease that manifests with syncope, most commonly in elderly patients. CSS affects around 1% of people with documented syncope [8]. CSS is classified into three different types, which include cardioinhibitory, vasodepressor, and a mixed form [8]. Cardioinhibitory CSS is characterized by bradycardia or three seconds of asystole. Vasodepressor type is a CSS caused by a reduction in systolic blood pressure by more than 50 mmHg. The mixed form presents as both bradycardia and decreased systolic blood pressure. Patients with either type of CSS may even experience syncope. In such cases, physicians should conduct a thorough exam to establish the cause of the CSS-induced syncope. Rarely, the presence of benign or cancerous tumors in the H&N region might induce CSS [3]. Since syncope can arise as the first sign of the disease, it is particularly challenging to manage in patients with H&N cancer.

We reported a case of CSS in a patient diagnosed with laryngeal cancer, which led to lymphedema and encasement of the bilateral carotid arteries. It is important to differentiate CSS from other causes of syncope with similar clinical presentations. Vasovagal syncope (VVS) is mainly triggered by emotional stress, pain, or prolonged standing, resulting in hypotension or bradycardia. It is typically preceded by feelings of lightheadedness, warmth, and nausea, with symptoms like tunnel vision, profuse sweating, and a ringing sound in the ears. VVS is more common in younger individuals, and management involves trigger avoidance and counter-pressure maneuvers [9]. Seizures are caused by abnormal electrical brain activity, leading to altered consciousness, involuntary movements, and postictal confusion, often with prodromal symptoms, such as aura or abnormal sensations [10]. Syncope due to cardiac arrhythmia results from abnormal heart rhythms, leading to inadequate cardiac output and cerebral perfusion, with symptoms like palpitations or chest pain preceding the syncopal episode [11]. 

The patient presented with an initial manifestation of syncope. During his hospital course, the patient experienced recurrent episodes of hypotension and bradycardia requiring atropine and transcutaneous pacemaker placement, suggesting a probable mixed type of CSS. The patient was discharged on long-term theophylline (xanthine) treatment to achieve symptomatic control of the bradycardia episodes. 

While there is currently no established therapy for CSS induced by malignant compression, various treatment strategies have been investigated for different forms of CSS. Treating vasodepressor CSS presents some challenges, yet initial management may involve reducing hypotensive medications and increasing fluid intake [12]. Fludrocortisone and midodrine have been used with some degree of success in patients with vasodepressor CSS [13]. Dual-chamber pacemakers have shown limited efficacy in vasodepressor CSS. One publication revealed that cardiac pacing failed in 3/3 of patients suffering from CSS due to the development of pure vasodepressor syncope [13]. 

Cardioinhibitory CSS has been treated through either pharmacological intervention or the implantation of dual-chamber pacemakers. Pharmacological management of cardioinhibitory CSS typically involves the administration of atropine, a cholinergic antagonist. Atropine inhibits vagus nerve stimulation via antagonism of acetylcholine receptors. In one particular study, a bolus dose of 700 mcg of atropine abolished the cardioinhibition of all patients suffering from carotid sinus hypersensitivity [14]. By contrast, dual-chamber pacemakers deliver electrical impulses to stimulate both atrial and ventricular contraction when the heart rate drops too low. An analysis of the benefits of pacemaker placement in patients affected by CSS revealed that syncope recurred in 57% of patients without a pacemaker, compared to only 9% of patients with a pacemaker [15]. Pacemaker placement also resulted in the resolution of the syncopal and seizure-like episodes in a similar case of a 62-year-old male suffering from CSS due to an invasive squamous cell carcinoma [7]. 

Despite promising outcomes in some cases of CSS treated with pacemakers, efficacy is not universal, and limitations may arise, particularly in H&N cancer patients. CSS induced by H&N cancer often resolves following cancer treatment [3]. While our patient remained asymptomatic on theophylline during radiation therapy, not all patients experience the same stability with pharmacological or pacemaker interventions. In one case, CSS persisted despite pacemaker placement but resolved with chemotherapy drugs such as cisplatin and docetaxel [16]. The conflicting evidence regarding the recurrence of syncopal episodes after pacemaker placement raises questions about its utility, especially considering its invasiveness and the potential for no longer needing it after cancer treatment. Therefore, our patient was discharged with theophylline rather than a pacemaker, considering the uncertainties and potential drawbacks associated with pacemaker placement in this context.

Although not widely employed, theophylline has shown to be effective in temporarily managing CSS in H&N cancer patients, eliminating the requirement for invasive pacemaker placement [17]. Theophylline acts as an adenosine receptor antagonist and phosphodiesterase inhibitor, thereby inhibiting the breakdown of cyclic AMP in smooth muscle cells. This mechanism reverses the effects of adenosine receptors and cyclic AMP on vagal manifestations. Following the administration of atropine and intravenous fluid during hospitalization, our patient's CSS was successfully managed with theophylline while he underwent cancer treatment.

The primary treatment approach for laryngeal cancer typically involves chemoradiotherapy and, if feasible, surgical resection. Common chemotherapy agents used for squamous cell carcinomas of the H&N include cisplatin, paclitaxel, docetaxel, 5-fluorouracil, and cetuximab [18]. However, chemoradiotherapy is associated with several side effects, including lymphedema. A study examining edema in oral and oropharyngeal cancer found that chemoradiation therapy led to an increase in posterior pharyngeal wall thickness, significantly impacting swallowing function [19]. In light of these findings, our patient initially underwent radiation therapy without chemotherapy to mitigate the risk of exacerbating his CSS-related edema. Following radiation therapy, his symptoms improved, although he continued to experience tachycardia. While edema poses a detrimental impact in many scenarios, its ramifications are particularly severe for individuals with H&N cancer, and thus, clinicians should extensively consider it when coordinating treatment plans. 

Despite its infrequency, CSS should be considered in patients who experience convulsive syncope alongside H&N malignancy. Due to the scarcity of evidence and consensus regarding CSS treatment in H&N cancer patients, additional research is necessary to compare and evaluate the available options. Moreover, newer treatment modalities may warrant exploration but will require further investigation. In 2023, the first case of cardioneuroablation for the management of CSS secondary to oropharyngeal squamous cell carcinoma was reported [20]. By combining traditional and innovative techniques, a more precise diagnostic approach should be established to enhance outcomes in these patients.

## Conclusions

Although uncommon, the occurrence of unexplained syncope should prompt clinicians to consider the possibility of carotid sinus compression by H&N cancers. Presently, treatment for such cases is complex due to their rarity. Various approaches may be pursued and should be tailored to each patient based on the frequency of vagal episodes, response to short-term management, and the nature of the cancer. Further research and case reports are necessary to establish a consensus on the optimal management approach for patients affected by CSS due to compression from H&N cancers.
